# Surgery for IDH1/2 wild-type glioma invading the corpus callosum

**DOI:** 10.1007/s00701-020-04623-z

**Published:** 2020-10-23

**Authors:** Pamela Franco, Daniel Delev, Debora Cipriani, Nicolas Neidert, Elias Kellner, Waseem Masalha, Bianca Mercas, Irina Mader, Peter Reinacher, Astrid Weyerbrock, Christian Fung, Jürgen Beck, Dieter Henrik Heiland, Oliver Schnell

**Affiliations:** 1 Department of Neurosurgery, Medical Center - University of Freiburg, Breisacher Straße 64, 79106 Freiburg, BW Germany; 2 Faculty of Medicine, University of Freiburg, Freiburg, BW Germany; 3 Department of Neurosurgery, University of Aachen, Aachen, NRW Germany; 4 Department of Radiology, Medical Centre - University of Freiburg, Freiburg, BW Germany; 5 Specialist Centre for Radiology, Schoen Clinic, Vogtareuth, BY Germany; 6 Department of Neurosurgery, Division Stereotactic and Functional Neurosurgery, Medical Center- University of Freiburg, Freiburg, BW Germany

**Keywords:** Corpus callosum glioblastoma, Butterfly glioblastoma, Glioblastoma, Neurosurgery, IDH status, Molecular diagnosis, Extent of resection

## Abstract

**Background:**

Glioblastoma of the corpus callosum (ccGBM) are rare tumors, with a dismal prognosis marked by a rapid clinical deterioration. For a long time, surgical treatment was not considered beneficial for most patients with such tumors. Recent studies claimed an improved survival for patients undergoing extensive resection, albeit without integration of the molecular profile of the lesions. The purpose of this study was to investigate the effect of biopsy and surgical resection on oncological and functional outcomes in patients with IDH wild-type ccGBM.

**Methods:**

We performed a retrospective analysis of our institution’s database of patients having been treated for high-grade glioma between 2005 and 2017. Inclusion criteria were defined as follows: patients older than 18 years, histopathological, and molecularly defined IDH wild-type glioma, major tumor mass (at least 2/3) invading the corpus callosum in the sagittal plane with a uni- or bilateral infiltration of the adjacent lobules. Surgical therapy (resection vs. biopsy), extent of resection according to the remaining tumor volume and adjuvant treatment as well as overall survival and functional outcome using the Karnofsky Performance Score (KPS) were analyzed.

**Results:**

Fifty-five patients were included in the study, from which the mean age was 64 years and men (*n* = 34, 61.8%) were more often affected than women (*n* = 21, 38.2%). Thirty (54.5%) patients were treated with stereotactic biopsy alone, while 25 patients received tumor resection resulting in 14.5% (*n* = 8) gross-total resections and 30.9% (*n* = 17) partial resections. The 2-year survival rate after resection was 30% compared to 7% after biopsy (*p* = 0.047). The major benefit was achieved in the group with gross-total resection, while partial resection failed to improve survival. Neurological outcome measured by KPS did not differ between both groups either pre- or postoperatively.

**Conclusions:**

Our study suggests that in patients with corpus callosum glioblastoma, gross-total resection prolongs survival without negatively impacting neurological outcome as compared to biopsy.

**Electronic supplementary material:**

The online version of this article (10.1007/s00701-020-04623-z) contains supplementary material, which is available to authorized users.

## Introduction

Glioblastomas are the most common malignant astroglial-derived tumors accounting for 47.1% of all central nervous system (CNS) tumors with an incidence of 3.20 per 100,000 persons per year [1]. Despite best available treatment, the median overall survival rate is about 16 months [2–5]. In general, glioblastoma treatment is based on attempting complete resection of contrast-enhancing tumor followed by adjuvant therapy [6]. However, treatment for glioblastomas initially infiltrating the corpus callosum remains controversial [7–9]. These tumors present with a characteristic growth pattern that invades more or less both cerebral hemispheres. Most commonly, they arise within the frontal lobe but can also be found in the parietal and occipital lobes. If both hemispheres are almost equally infiltrated, they are referred to as butterfly glioblastomas [8, 10, 11]. Clinically, these tumors have a wide range of symptomatic presentations including disorientation, focal epilepsy as well as comatose, and mute states [8, 11]. Since these tumors invade eloquent areas, complete resection is often unlikely. Furthermore, several authors argue that even the attempt of gross-total resection would be too aggressive and may lead to a more rapid clinical deterioration and delay adjuvant treatment [8, 9]. Therefore, until lately, diagnostic and molecular profiling via tumor biopsy was widely favored over the attempt of gross-total tumor resection. Yet, a growing number of authors have published data highlighting the benefits of resection of glioblastoma of the corpus callosum (ccGBM), specifically regarding improved overall survival without aggravating persistent neurological deficits [12–14]. Unfortunately, there is a lack of consistent molecular profiling of the tumors in the previous studies, which leads to a disputable interpretation of the benefits of surgical resection upon survival in this subset of tumors. An IDH mutation is an independent prognostic factor that improves overall survival (OS) in glioblastoma [15]. The aim of this study was to explore the impact of tumor resection and adjuvant treatment strategies on IDH wild-type corpus callosum glioblastoma.

## Materials and methods

### Patient data acquisition

We screened all patients treated for malignant brain tumors at the Department of Neurosurgery at the Medical Center-University of Freiburg between 2005 and 2017. Eligibility criteria were a suspicion of corpus callosum glioblastoma on MR imaging, later confirmed by histopathology and molecular profiling, on adult patients (older than 18 years of age). Patients with multilocular glioblastoma (i.e., infiltration of more than three lobes) as well as patients with glioblastoma manifestation distant from the corpus callosum were excluded from the study. Patient demographics are listed in Table 1. Clinical data were extracted from medical archives. Written informed consent for utilization of clinical data for scientific purposes was obtained preoperatively from all patients. The local ethics committee of the University of Freiburg approved data evaluation (protocol 472/15).

**Table 1:** Patient demographics

	Entire cohort		Surgical resection		Biopsy		
	*N*	%	*N*	%	*N*	%	*p*
Patients	55	100	25	45.5	30	54.5	-
Gender							
Female	21	38.2	10	40	11	36.7	0.82
Male	34	61.8	15	60	19	63.3	0.82
Age in years							
Median (IQR)	66	(57.5–73)	62.3	(50.5–66.9)	68.5	(62.1–76)	-
Symptomatic at first presentation							
Memory impairment	15	27.3	3	12	12	40	0.02
Confusion/mental status alteration	38	69	17	68	21	70	0.87
Visual field deficit	6	11	4	16	2	6.7	0.27
Aphasia	10	18.2	7	28	3	10	0.08
Cranial nerve impairment	2	3.6	1	4	1	3.3	0.89
Motor function impairment	13	23.6	7	28	6	20	0.49
Sensory function impairment	3	5.5	1	4	2	6.7	0.66
Psychiatric alteration	1	1.8	0	-	1	3.3	0.36
Headache	7	12.7	6	24	1	3.3	0.01
Epilepsy	11	20	3	12	8	26.7	0.20

*IQR*, interquantile range

### Histopathological and molecular diagnostics

Specimens were fixed in 4% phosphate-buffered formaldehyde and paraffin-embedded according to standard procedures in the Institute of Neuropathology, Medical Center-University of Freiburg, as detailed described in our previous works [16, 17]. In order to determine the IDH mutation status, we performed IHC (R132H). In patients younger than 65 years, we used next-generation sequencing of IDH1 and IDH2 to confirm negative staining results.

### MR imaging acquisition

Imaging was performed on 1.5-T and 3-T whole body system (Siemens Magnetom Avanto, Trio, or Prisma, Erlangen, Germany). Anatomical imaging consisted of 3D T1-weighted sequences (MPRAGE, TR 2300 ms, TI 988 ms, TE 2.26 ms, voxel size 1 mm^3^) before and after contrast application (0.1 mM gadoteridol per kg body weight (ProHance®, Bracco, Konstanz, Germany)) and 2D T2-weighted turbo spin-echo sequence (TR 4500 ms, TE 100 ms, echo train length 17, voxel size 0.6 × 0.6 × 2 mm^3^). Patients received a postoperative MRI scan with and without contrast enhancement within the first 72 h after surgical resection and regular MRI scans every 3 months from then on. Tumor progression was defined according to RANO-criteria [18]. Extent of resection was assessed on pre- and postoperative MRI; gross-total resection was defined when less than 5% remaining tumor was detected [19].

### Tumor segmentation

From the patients with complete and qualitatively acceptable 3D MR imaging (*n* = 36), tumor segmentation was performed. First, images were converted to a NifTI format and loaded into a Web-based framework for medical imaging analysis (http://www.nora-imaging.com). Segmentation was then carried out by a semi-automated approach and processed in R-software. Tumor volumetry was performed based on the contrast-enriched tumor region.

### Surgical treatment

Groups were divided based on the main treatment strategy (gross-total or partial resection and stereotactic biopsy). Patients who underwent surgical resection within the first month after stereotactic biopsy were assigned to the resection group.

### Oncological treatment

Patients received glioblastoma-specific oncological treatment according to the protocol defined by Stupp et al. in 2005 whenever patients consented to the treatment and if they had a good clinical status (Karnofsky Performance Scale (KPS) > 70%). Alternative treatments, i.e., either chemotherapy with temozolomide or lomustine alone or radiotherapy alone, were also present in the study.

### Primary endpoint

The primary endpoint was overall survival after first diagnosis, which was defined as the time until death from any cause (patients with event) or until last contact (patients without event). Secondary endpoints were surgical and neurological outcomes. For the latter, we used the KPS and clinical parameters.

### Statistical analysis

We used the Kaplan-Meier method to estimate survival distributions and a Cox proportional-hazards model to calculate hazard ratios. Statistical power was measured by “powerSurvEpi,” an R-software package. With a calculated power over 80%, we set the alpha level to 5% (*p* < 0.05) for statistical significance. Patients lost at follow-up where censored at the recorded date of last contact. Numeric variables were tested for normal distribution by Shapiro-Wilks test. In case of a normal distribution, variables were represented by mean and standard deviation and tested by non-paired Student’s *t* test. In case of non-normal distribution, variables were presented with median and interquantile range (IQR), tested by Wilcoxon rank sum test. Nominal variables were tested by either chi-square or Fisher’s exact test.

## Results

### Patients’ demographics

A total of 2720 patients were treated for high-grade glioma between 2005 and 2017 at the Medical Center-University of Freiburg. Fifty-five adult patients with molecularly defined IDH wild-type glioma of the corpus callosum were included in the study. Median age was 66 years (IQR 57.5–73). Male patients accounted for 61.8% (*n* = 34) of the cases, while females represented 38.2% (*n* = 21). The leading clinical symptom at first presentation was confusion/alteration of mental status in 69% of patients followed by memory impairment (27.3%), motor function impairment (23.6%), and epilepsy (20%). Other clinical symptoms included aphasia (18.2%), headache (12.7%), and visual field deficit (11%) and less common were sensory function impairment (5.5%), cranial nerve impairment (3.6%), and psychiatric alteration (1.8%). Regarding the anatomical invasion of the corpus callosum, the genu was significantly more often invaded (*p* = 0.005).

### Characteristics of the biopsy and resection cohorts

Thirty patients (54.6%) received a biopsy to confirm the histology from which 50% were further treated by chemotherapy alone (*n* = 6, 20%) or combined radiotherapy plus chemotherapy (*n* = 9, 30%). Twenty-five patients were treated by surgical resection from which 8 patients received a gross-total resection, and 17 patients were partially resected. The decision towards resection over biopsy was mainly driven by high tumor burden and mass effect of the tumor which was confirmed by our volumetric evaluation. The mean volume in the group of resected patients was significantly greater compared the biopsy group (resection: 59.4 cm^3^, biopsy 35.2 cm^3^, *p* = 0.01). Postoperative volume after resection was decreased by tenfold (mean volume: 5.96 cm^3^). The mean postoperative tumor volume in the biopsy group remained constant. Received adjuvant treatment was well balanced in both groups regarding treatment modalities and duration of the treatment. In our cohort, we found that gliomas localized in the genu were preferentially resected in comparison to other anatomical locations (resection *n* = 17 vs. biopsy *n* = 9, *p* = 0.005), Table 2.

**Table 2:** Surgery characteristics

	Entire cohort		Surgical resection		Biopsy		
	*N*	%	*N*	%	*N*	%	*p*
Patients	55	100	25	45.5	30	54.5	-
Site of CC invasion							
Rostrum	1	1.8	0	0	1	3.3	0.36
Genu	26	47.2	17	68	9	30	0.005
Genu/body	5	9	2	8	3	10	0.79
Body	11	20	3	12	8	26.7	0.18
Body/splenium	6	11	1	4	5	16.7	0.13
Splenium	6	11	2	8	4	13.3	0.53
Surgery							
GTR	8	14.55					
PR	17	30.9					
Biopsy	30	54.55					
Tumor volume in cm^3^							
Preoperative median	43.5		59.4		35.2		0.01
Postoperative median	5.96		5.96		35.2		*
Complication							
None	42	76.3	17	68	25	83.3	0.18
Hemorrhage	3	5.4	3	12	0	0	0.05
Ischemia	1	1.8	1	4	0	0	0.27
Cranial nerve deficit	1	1.8	1	4	0	0	0.27
Meningitis	2	3.6	2	8	0	0	0.84
Hydrocephalus	3	5.4	1	4	2	6.7	0.81
Aphasia	3	5.4	1	4	2	6.7	< 0.001
Pneumonia	1	1.8	0	0	1	3.3	0.89

*IQR*, interquantile range; *CC*, corpus callosum; *GTR*, gross-total resection; *PR*, partial resection

### Follow-up outcome

Regarding functional outcome, we found that patients in both the surgery and biopsy groups had an improvement of their KPS immediately postoperative, and in the first 2 weeks after the hospital stay, this is most probably due to the effect of steroid treatment perioperatively. The surgery group contained 52.9% of all patients with a KPS below 80% before hospitalization, which improved to only 33.3% patients with a KPS below 80% after hospitalization. In the biopsy group, patients with a KPS under 80% also saw a similar reduction from 69.6 to 33.3% after hospital stay (Table 3). A significant difference between both surgery and biopsy was not detected. At the 3- and 12-month follow-up, the number of patients with a KPS below 80% was constantly increasing in the biopsy group (KPS < 80% at 3 months: 82%, at 12 months: 92%) and the partial resection group (KPS < 80% at 3 months: 86.6%, at 12 months: 86.6%). The only significant difference was found in the gross-total resection group, where 60% of the patients maintained a KPS over 80% at 3-month follow-up (*p* = 0.027). At 12-month follow-up, only 5 patients presented with a KPS over 80%; here, no significant difference between biopsy or surgery was detected (Table 4).

**Table 3:** Oncology characteristics

	Entire cohort		Surgical resection		Biopsy		
	*N*	%	*N*	%	*N*	%	*p*
Patients	55	100	25	45.5	30	54.5	-
Adjuvant treatment							
RCT	16	29.1	7	28	9	30	0.87
CT	13	23.4	7	28	6	20	0.49
RT	3	5.5	3	12	0	0	0.05
Palliative	23	41.8	8	32	15	50	-
MGMT-promoter status							
Methylated	15	27.3	4	16	11	36.6	0.09
Unmethylated	15	27.3	9	36	6	20	0.18
NA	25	45.4	12	48	13	43.4	-

*RCT*, radiochemotherapy; *CT*, chemotherapy; *RT*, radiotherapy; *NA*, not available

**Table 4:** Karnofsky Performance Score

	Entire cohort		Surgical Resection		Biopsy		
	*N*	%	*N*	%	*N*	%	*p*
Patients	55	100	25	45.5	30	54.5	-
Preoperative							
100	0	-	0	-	0	-	-
90	17	30.9	10	40	7	23.3	0.18
80	23	41.8	8	32	15	50	0.18
< 80	15	27.3	7	28	8	26.7	0.91
Postoperative							
100	13	23.6	9	36	4	13.3	0.05
90	22	40	8	32	14	46.7	0.27
80	6	10.9	4	16	2	6.7	0.27
< 80	14	25.5	4	16	10	33.3	0.14
3-month follow-up							
100	5	9.1	4	16	1	3.33	0.10
90	7	12.7	3	12	4	13.33	0.88
80	6	10.9	2	8	4	13.33	0.53
< 80	12	21.8	7	28	5	16.7	0.31
0	25	45.5	-	-	-	-	-
12-month follow-up							
100	4	7.3	2	8	2	6.7	0.85
90	1	1.8	1	4	0	-	0.27
80	0	-	-	-	-	-	-
< 80	10	18.2	7	28	3	10	0.08
0	40	72.7	-	-	-	-	-

### Surgical complications

Thirteen patients (23.6%) had postoperative complications. In the surgical resection group, the leading complications were postoperative hemorrhage in the resection cavity (*n* = 3) and meningitis (*n* = 2). Other complications included territorial infarction leading to decompressive hemicraniectomy, cranial nerve deficit, hydrocephalus requiring emergency external ventricle drainage placement, and aphasia (*n* = 1 each). In the biopsy group, 2 patients developed postoperative hydrocephalus requiring emergency external ventricle drainage placement, 2 patients developed aphasia, and one patient had an acquired bacterial pneumonia requiring intensive care. All other clinical features remained well balanced between both groups; clinical characteristics are listed in Table 1 and surgical characteristics are listed in Table 2.

### Outcome analysis of biopsy vs. resection

**Fig. 1:**
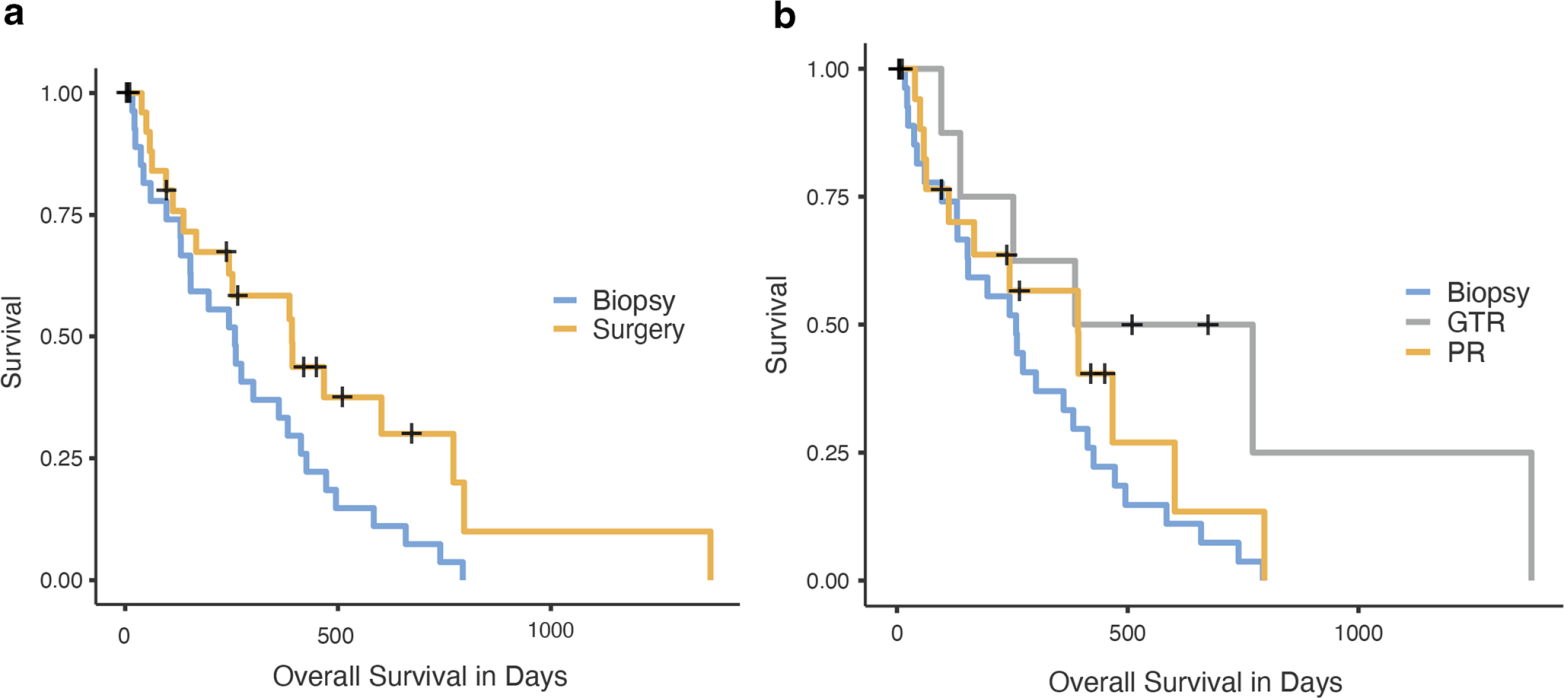
**a** Kaplan-Meier analysis of the impact of surgical resection vs. biopsy on survival. **b** Kaplan-Meier curve for OS stratified by type of surgical approach: gross-total resection, partial resection, and biopsy. GTR, gross-total resection; PR, partial resection

**Fig. 2:**
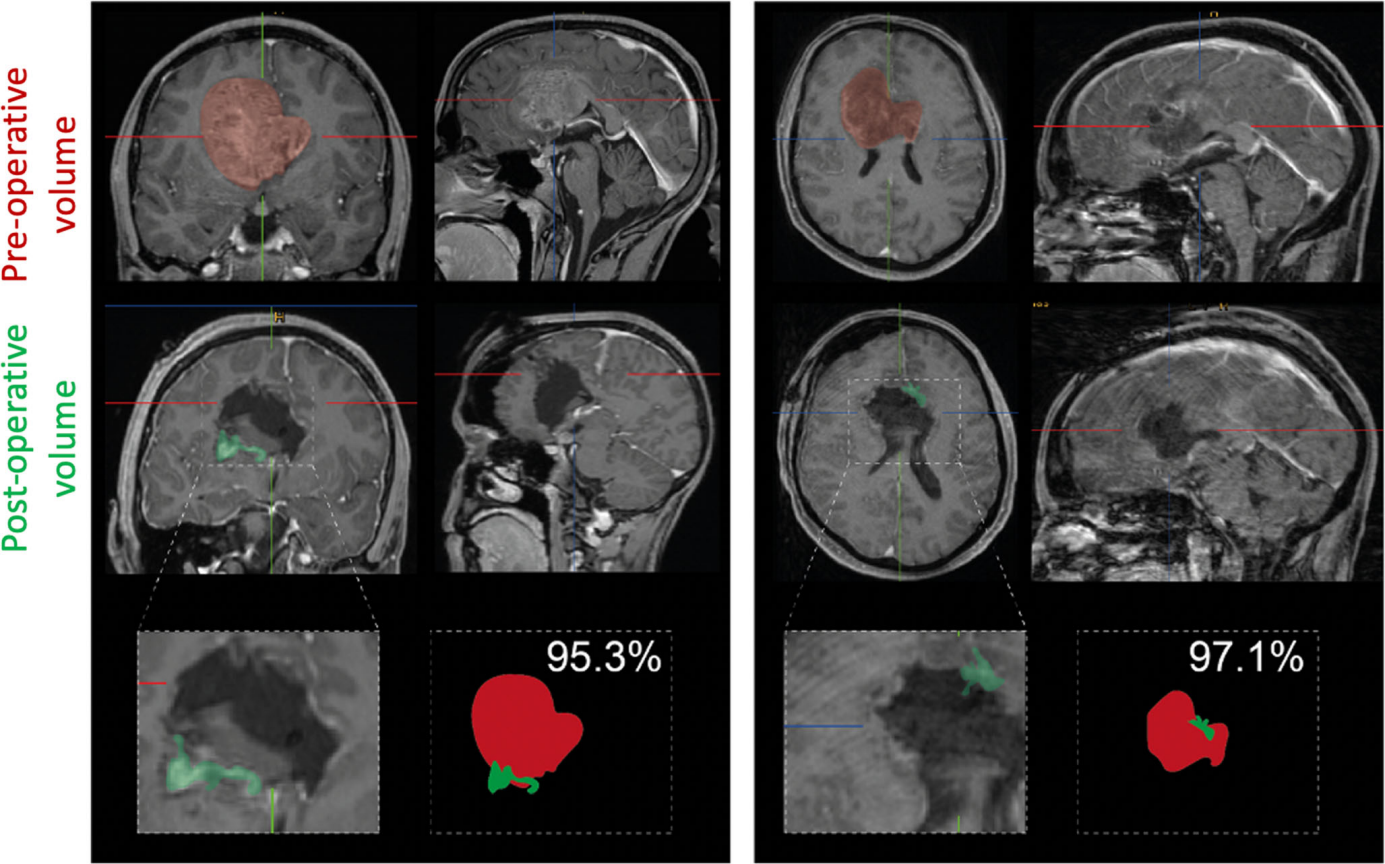
Two examples of patients with glioblastoma invasion of the corpus callosum who received a gross-total resection; the preoperative (red) as well as the postoperative (residual, green) tumor volumes are illustrated

We analyzed the effect of a surgical resection on overall survival (primary outcome). Forty-five patients reached the defined endpoint (resection *n* = 18, biopsy *n* = 27), while 10 patients were censored (resection *n* = 7, biopsy *n* = 3). We used a Cox proportional-hazards model to estimate the outcome differences, which resulted in a significantly improved survival for patients who underwent a resection (258 days CI 95% 153–413 days) compared to those biopsied (220 days CI 95% NA), Fig. 1. The difference of the median survival was found to be relatively small, but the 2-year survival rate was increased from 7% of biopsy patients to 30% in the resection group (*p* = 0.047). In order to confirm our findings, we used a multivariate approach by Cox proportional-hazards model including other outcome relevant parameters such as the presence of epilepsy, the anatomical location of the tumor, the type of adjuvant treatment, age, and KPS (Table 6); the impact of a resection remained significant. In line with the literature in glioblastoma, age was a significant factor upon survival. We further analyzed to what degree the extent of a surgical resection impacted outcome. We found that partial resection did not significantly improve survival, whereas gross-total resection did pose a clear benefit on survival compared to biopsy, which translates into a better outcome for the surgical resection group on the whole (Table 5, Table 6, Fig. 1). Thus, even if 2 out of 3 patients do not receive a gross-total resection due to functional limitations in eloquent areas, the cohort of resected patients still benefits from the mass reduction (Fig. 2).

**Table 5:** Survival analyses

	Entire cohort		Surgical resection		Biopsy		
	*N*	%	*N*	%	*N*	%	*p*
Patients	55	100	25	45.5	30	54.5	-
Median OS (CI 95%)	252 (123–513)		258 (153–413)		220 (244–NA)		0.041
Median PFS	153		146		154		
3-month survival	42	76	21	84	21	78	n.s.
6-month survival	32	58	16	67	16	59	n.s.
12-month survival	21	38	12	58	9	33	0.032
24-month survival	5	9	3	30	2	7	0.047

*OS*, overall survival; *PFS*, progression-free survival; *n.s.*, not significant

**Table 6:** Multivariate Cox regression analyses

Multivariate Cox regression			
	HR	CI 95%	*p* value
Surgical treatment			
Biopsy	*		
GTR	0.35	0.13–0.93	*0.036*
PR	0.76	0.38–1-52	0.431
Adjuvant treatment			
CT	*		
Palliative	2.77	1.20–6.39	*0.017*
RCT	0.81	0.34–1.93	0.629
RT	1.57	0.31–7.91	0.586
Anatomy			
Genu	*		
Splenium	0.41	0.12–1.42	0.160
Sex			
Male	*		
Female	1.00	0.53–1.90	0.997
Epilepsy	0.70	0.33–1.51	0.369
Age			
Continuous variable	1.05	1.01–1.08	0.013
KPS			
Ordinal variable	0.99	0.96–1.02	0.396

Significant *p-values* are shown italicized

*HR*, hazard ratio; *CI*, confidence interval; *GTR*, gross-total resection; *PR*, partial resection; *KPS*, Karnofsky Performance Score

*Reference variable

## Discussion

### Treatment of ccGBM—still a matter of debate

Corpus callosum glioblastomas are rare tumors invading a highly eloquent area of the brain, which results in a difficult treatment decision-making process and argues for a personalized approach. The decisive challenge of treating eloquent tumors lies within the delicate balance between avoiding further neurological impairment and relieving already existing ones. Since a pronounced mass effect of the tumor may rapidly lead to severe neurological deterioration, tumor resection should not be ruled out in general. Meanwhile, an optimal neuro-oncological therapy regime is also required to achieve the maximal survival benefit for the patient. In our study, we sought to address this dilemma and to determine to what extent different treatment strategies affect the neurological and oncological outcomes. Our data showed that the major factor which drives the decision towards a surgical removal of the tumor was mass effect resulting in a significantly larger tumor volume in the resection cohort, which we tested using a generalized linear model (Supplementary Table 1). We were able to show that this mass effect can be effectively treated with a calculated risk and without postoperative loss of functional performance. This observation shows an important limitation of our retrospective study because large tumors were never treated by biopsy and adjuvant treatment only due to a high risk of rapidly progressive neurological deterioration and the associated inability to receive adjuvant treatment. The importance of cytoreduction was also reported by Chaichana et al. who was able to show that mass reduction causes favoral neurological outcome and leads to improved functional performance even if the surgical removal comes with higher risk of surgery-associated complications.

### Surgical resection improves survival outcome without increasing neurological morbidity

In line with other authors, we showed a survival benefit for patients who underwent tumor resection [12–14]. However, compared to other published works, the extent of survival benefits is distinctly lower in our cohort. This is due to the improved outcome of the biopsy group. By comparison, already published data indicates a survival of non-resected patients between 1.3 and 3.5 months [12–14, 20]. In contrast, our patients from the biopsy group achieved a median survival of 7.2 months, which is a substantial increase compared to previous reports. This benefit results from a consequent adjuvant treatment of most of the patients, mostly by combined radio- and chemotherapy, on the one hand, and the high percentage of lacking adjuvant therapy in comparable studies, on the other hand. Noteworthy, tumor resection improved patients’ survival, but did not lead to an increased rate of neurological deficits. Moreover, patients undergoing resection showed better neurological outcome 1 year after treatment compared to biopsied patients. This result could be explained by the fact that the gross-total resections succeeded in reducing tumor volume by more than 95%. This consequently resulted in a meaningful reduction of tumor-associated edema and mass effect–associated symptoms.

### Strengths and limitations

Compared to other studies, our cohort was defined by integrative histopathology including determination of the IDH1/2 mutation by immunohistochemistry (IHC) or next-generation sequencing (NGS). From many recent studies, we have learned that mixed histology causes strong bias and misinterpretation of outcome data. Here, we avoid this bias by excluding all patients without clearly defined IDH status. However, a limitation of our study was the reduced number of patients who also had a defined MGMT-Promoter status, due to an uneven integration in the diagnostic work-up at our institution until 2016 (only patients older than 70 years received this assessment routinely).

The cohort of corpus callosum glioma patients is rather small in all previously reported studies and ranges between 29 and 39 cases per institute. Within similar time periods, the number of patients treated at our university was 55. Although our work is based on retrospective analysis, it validates existing findings and supports empirical evidence.

Although ccGBM accounts for only a small percentage of malignant brain tumors, a prospective study which specifically addresses the impact of different treatment modalities on quality of life and neuropsychological and social aspects is necessary to further improve decision-making in these patients.

## Conclusion

Our study aimed to analyze the impact of tumor resection in the largest cohort of IDH wild-type glioma in the corpus callosum (ccGBM) described in the literature thus far. We confirmed that resection of ccGBM is feasible and significantly improves overall survival. In accordance with the common practice of maximum resection in glioblastoma, gross-total resection can be achieved without necessarily leading to aggravation of neurological status. Furthermore, even improvement of functional outcome was possible in gross-total-resected patients compared to biopsied patients at 3-month follow-up. Therefore, surgical resection may be associated with risks, but should be taken into consideration in the management of ccGBM.

## Electronic supplementary material

ESM 1 (DOCX 14 kb)

## Abbreviations

ccGBM: Glioblastoma of the corpus callosum
IDH: Isocitrate dehydrogenase
CNS: Central nervous system
KPS: Karnofsky Performance Score
OS: Overall survival
MR: Magnetic resonance
RANO: Response Assessment in Neuro-Oncology criteria
