# Supplementary material for: Surgery for IDH1/2 wild-type glioma invading the corpus callosum
Source: Acta Neurochir (Wien). 2020 Oct 23;163(4):937–45. doi: 10.1007/s00701-020-04623-z (PMC7966629; doi:10.1007/s00701-020-04623-z)
Supplement: Supplementary file 1 — (DOCX 14 kb) [file 701_2020_4623_MOESM1_ESM.docx]

Supplementary Table 1

|  | | | | | | | | | | | | | | | | | | | | | |
| --- | --- | --- | --- | --- | --- | --- | --- | --- | --- | --- | --- | --- | --- | --- | --- | --- | --- | --- | --- | --- | --- |
|  | | | | | | | | **95% Confidence Interval** | | | |  | | **95% Exp(B) Confidence Interval** | | | |  | | | |
| **Names** | | **Effect** | | **Estimate** | | **SE** | | **Lower** | | **Upper** | | **exp(B)** | | **Lower** | | **Upper** | | **z** | | **p** | |
| Age |  | Age |  | -0.00393 |  | 0.0331 |  | -0.07400 |  | 0.0601 |  | 0.996 |  | 0.929 |  | 1.06 |  | -0.119 |  | 0.906 |  |
| Gender |  | Male - Female |  | 0.38665 |  | 0.8035 |  | -1.17395 |  | 2.0359 |  | 1.472 |  | 0.309 |  | 7.66 |  | 0.481 |  | 0.630 |  |
| KPS |  | KPS |  | 0.00332 |  | 0.0303 |  | -0.05898 |  | 0.0651 |  | 1.003 |  | 0.943 |  | 1.07 |  | 0.110 |  | 0.913 |  |
| **Tumor volume** |  | **Tumor volume** |  | **0.03476** |  | **0.0154** |  | **0.00749** |  | **0.0693** |  | **1.035** |  | **1.008** |  | **1.07** |  | **2.262** |  | **0.024** |  |
|  | | | | | | | | | | | | | | | | | | | | | |
